# Steroid receptor coactivator TAIMAN is a new modulator of insect circadian clock

**DOI:** 10.1371/journal.pgen.1010924

**Published:** 2023-09-08

**Authors:** Vlastimil Smykal, Lenka Chodakova, Marketa Hejnikova, Kristina Briedikova, Bulah Chia-Hsiang Wu, Hana Vaneckova, Ping Chen, Anna Janovska, Pavlina Kyjakova, Martin Vacha, David Dolezel

**Affiliations:** 1 Biology Centre of the Academy of Sciences of the Czech Republic, Institute of Entomology, Ceske Budejovice, Czech Republic; 2 Faculty of Science, University of South Bohemia, Ceske Budejovice, Czech Republic; 3 Faculty of Science, Masaryk University, Brno, Czech Republic; 4 Institute of Organic Chemistry and Biochemistry of the Czech Academy of Sciences of the Czech Republic, Prague, Czech Republic; Universidad de Valparaiso, CHILE

## Abstract

TAIMAN (TAI), the only insect ortholog of mammalian Steroid Receptor Coactivators (SRCs), is a critical modulator of ecdysone and juvenile hormone (JH) signaling pathways, which govern insect development and reproduction. The modulatory effect is mediated by JH-dependent TAI’s heterodimerization with JH receptor Methoprene-tolerant and association with the Ecdysone Receptor complex. Insect hormones regulate insect physiology and development in concert with abiotic cues, such as photo- and thermoperiod. Here we tested the effects of JH and ecdysone signaling on the circadian clock by a combination of microsurgical operations, application of hormones and hormone mimics, and gene knockdowns in the linden bug *Pyrrhocoris apterus* males. Silencing *taiman* by each of three non-overlapping double-strand RNA fragments dramatically slowed the free-running period (FRP) to 27–29 hours, contrasting to 24 hours in controls. To further corroborate TAIMAN’s clock modulatory function in the insect circadian clock, we performed *taiman* knockdown in the cockroach *Blattella germanica*. Although *Blattella* and *Pyrrhocoris* lineages separated ~380 mya, *B*. *germanica taiman* silencing slowed the FRP by more than 2 hours, suggesting a conserved TAI clock function in (at least) some insect groups. Interestingly, the pace of the linden bug circadian clock was neither changed by blocking JH and ecdysone synthesis, by application of the hormones or their mimics nor by the knockdown of corresponding hormone receptors. Our results promote TAI as a new circadian clock modulator, a role described for the first time in insects. We speculate that TAI participation in the clock is congruent with the mammalian SRC-2 role in orchestrating metabolism and circadian rhythms, and that TAI/SRCs might be conserved components of the circadian clock in animals.

## Author summary

Most living creatures need to synchronize their rhythmic biological functions with the 24-hour day/night cycle of Earth. Instead of pocket watches or cell phones, they use intracellular molecular clocks to anticipate the daily recurring changes. Those circadian clocks are at the genetic level remarkably conserved among mammals and insects. Here, for the first time in insects, we introduce the protein TAIMAN as a new modulator of the circadian clock. The removal of TAIMAN from the adult linden bug *Pyrrhocoris apterus* or the German cockroach *Blattella germanica* males slows the clock by 2–4 hours. Circadian clocks often adjust animals’ physiology through various hormones and neuropeptides. Insect endocrinology is dominated by the steroid and steroid-like hormones, ecdysone, and juvenile hormone: and TAIMAN is closely engaged in both juvenile hormone and ecdysone receptor signaling. However, the circadian role of TAIMAN presented here modulates the clock in a hormone-independent way. Importantly, TAIMAN is related to the mammalian SRC-2 protein, which is involved in regulating circadian clock machinery in mice. Thus, the novel role we describe for TAIMAN in insects may have a counterpart in mammalian physiology.

## Introduction

Circadian clocks are endogenous oscillators that synchronize multiple physiological and behavioral processes with the day/night cycle. The central insect pacemaker in the brain is a coupled neuronal network creating oscillation and rhythmicity by using several transcriptional-translational interlocked molecular feedback loops (TTFL) [1]. In *Drosophila*, transcriptional activators CIRCADIAN LOCOMOTOR OUTPUT CYCLES KAPUT (CLOCK, CLK) [2] and CYCLE (CYC) [3], an insect homolog of mammalian BRAIN AND MUSCLE ARNT-LIKE 1 (BMAL1), bind promotors of negative regulators *period* (*per*) and *Drosophila*-type *timeless* (*d-tim*) and activate their transcription. Upon transfer to the cytoplasm, *per* and *d-tim* mRNA get translated, and proteins, upon phosphorylation, enter the nucleus and inhibit their own transcription by binding to CYC and CLK [4].

CLK in the center of the clock is a target of multiple regulations. Two basic leucine zipper (bZIP) transcription factors, PAR DOMAIN PROTEIN 1 (PDP1) and VRILLE (VRI) activate or repress, respectively, *Clk* transcription and their transcription is in return activated by CLK/CYC [5]. Concurrently, CLK-CYC drives the expression of the basic helix-loop-helix (bHLH) transcription factor *clockwork orange* (*cwo*). CWO protein enhances the removal of CLK-CYC from the *per* and *d-tim* promoters to maintain transcription repression [6].

The complexity of the mammalian circadian clock is high due to duplications of some core circadian genes, such as *period* (*per1-3)* and mammalian-type *cryptochrome* (*m-cry1-2*, in the mammalian literature often abbreviated as *cry1*, *cry2*) and extensive interconnection with metabolism modulators including Steroid Receptor Coactivators [7,8]. The circadian clock architecture in insects is remarkably similar to the mammalian system, depending on a similar set of genes [9]. However, group-specific differences have accumulated through evolution, and thus comparative studies on various insect taxa with different clock setups can help unveil the plasticity and functionality of the insect clock. For example, cyclorrhaphan Diptera, including the fruit fly *Drosophila melanogaster*, is the only group that lost mammalian-type CRYPTOCHROME (m-CRY) [10]. The fruit fly circadian clock relies on *d-tim* in the repressive feedback loop [11,12], and d-TIM temporal degradation is a primary way of clock synchronization by light [13,14]. Moreover, *Drosophila*-type CRYPTOCHROME (d-CRY), lost in all Chordata, in *Drosophila* resets the circadian clock by serving as a neuronal blue light photoreceptor [15,16]. The honeybee *Apis mellifera* and other Hymenoptera use a more ‘mammalian-like’ circadian clock: they have lost *d*-*cry* and *d-tim* genes. Other insects, such as the linden bug *Pyrrhocoris apterus* (Heteroptera), combine mammalian and *Drosophila* clock setup with preserved *d-tim* but lost *d*-*cry* and thus enabled to study evolutionary transition clock state [10].

Insects’ physiological processes at the organismal level are to a great extent synchronized by the steroid hormone ecdysone and sesquiterpenoid juvenile hormone (JH) [17]. Both hormones have a similar way of action. JH binds to its receptor MET, encoded by the *Methoprene-tolerant* gene, and thus facilitates its heterodimerization with TAIMAN (TAI), an ortholog of mammalian steroid receptor coactivator, and drives the expression of target genes to regulate development and reproduction [18–22]. Ecdysone binds ECDYSONE RECEPTOR (EcR), heterodimerizes with its partner ULTRASPIRACLE (USP) [23], and upon replacement of corepressor SMRTER [24] by transcriptional coactivator TAIMAN, activates expression of target genes [25].

The potential crosstalk of the circadian clock and the humoral regulation by JH and ecdysone is reported at different regulatory levels but remains largely elusive. The release of ecdysone in some species display circadian timing [26–29] and Kumar et al. showed EcR expression in *Drosophila* clock neurons and established ecdysone-induced nuclear receptor (NR) *Ecdysone-induced protein 75B* (*E75*/*Eip75*), an insect homolog of mammalian repressor REV-ERB, as a direct repressor of *Clk* expression [30]. In mammals, REV-ERB is opposed by mammalian activator Retinoic Acid Receptor-Related Orphan Receptor α (RORα) [31,32] to keep circadian rhythms. E75B and NR HORMONE RECEPTOR 3 (HR3), an insect homolog of RORα, influence the cycling of *Clk*, *cyc*, and *d-tim* in the ametabolous insect *Thermobia domestica* [33]. Interestingly, not HR3 but NR HORMONE RECEPTOR 51 (HR51, also known as UNFULFILLED) was recruited in *Drosophila* to co-regulate *per* transcription [34], and together with E75B constitutes a TTFL. Further, the ecdysone-inducible ABC transmembrane transporter encoded by *Early gene at 23* (*E23*) facilitates lipophilic ecdysone transmembrane transport from the cell, and thus represses ecdysone signaling [35,36]. *E23* transcription is also regulated by CLK/CYC complexes, and *E23* knockdown increases *vri* expression [36].

Clock crosstalk with JH signaling is more unclear. Shin et al. [37] described the physical interaction of MET, CYC, and TAI (called FISC) in a female mosquito *Aedes aegypti* fat body. In their experiments, the presence of CYC in the JH receptor complex facilitated the circadian expression of JH target genes in the fat body peripheral clock. Surgical removal of *corpora allata*, a JH-producing gland, reciprocally changed the transcription of *per* and circadian clock target gene *Pdp1* in *P*. *apterus* fat body [38]. Further experiments demonstrated that JH acts through MET (but not TAI) together with CLK and CYC in the *P*. *apterus* gut to maintain the linden bug reproductive state by controlling reciprocal circadian expression of *Pdp1*_*iso1*_ and *m-cry* genes [39].

Although there are examples of the interaction of circadian proteins with JH and ecdysone signaling pathways, direct involvement of core JH and ecdysone receptors’ proteins in the central circadian clock remains elusive. To clarify this important gap, we used a combination of microsurgical and reverse genetic tools available to the linden bug, and addressed the role of JH and ecdysone signaling in a system where the developmental role can be clearly separated from the function in the fully formed adult organism. We identified steroid receptor coactivator *taiman* as a new insect clock gene where TAIMAN modulates the pace of the circadian clock in the linden bug *P*. *apterus* and German cockroach *B*. *germanica* and *tai* silencing dramatically slowed down clock free-running period in both species by 2–4 hours, while sustaining the clock rhythmic. Interestingly, the effect on the clock seems to be ecdysone- and JH-independent.

## Results

### *taiman* knockdown dramatically slows the pace of *Pyrrhocoris apterus* circadian clock in a juvenile hormone-independent manner

#### Identification of *taiman* isoforms

**Fig 1:**
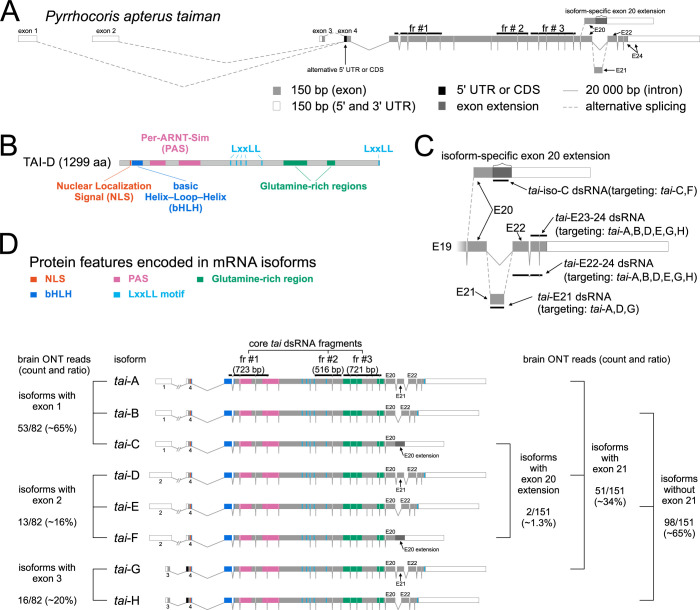
*P*. *apterus taiman* gene model, the position of RNA interference fragments and mRNA isoforms ratio in brain ONT transcriptomes. (A) *taiman* transcription starts from alternative exons 1, 2, and 3, which join to exon 4, read in two alternative frames. Exon 21 is alternatively spliced and exon 20 can be extended in an isoform-specific manner. Note the different scales of exons and introns. (B) Domain structure of *P*. *apterus* TAIMAN protein, represented by (longest) protein TAI isoform D (1299 amino acids). N-terminal DNA-binding basic-Helix-Loop-Helix (bHLH, blue) domain is followed by two Per-Arnt-Sim (PAS, pink) domains, five centrally located LxxLL motifs (light blue), C-terminal Glutamine-rich regions (green). (C) Positions of *taiman* isoform-specific RNA interference fragments. Both *tai*-E21 and *tai*-iso-C target single exons, *tai*-E23-24 and *tai*-E22-24 target two and three exons, respectively. (D) All eight detected *P*. *apterus taiman* transcripts with color-highlighted exons encoding protein domains and motifs. Three core *taiman* RNA interference fragments mapped to *tai*-A transcript target all *tai* isoforms. Semi-quantitative expression of *tai* isoforms/exons. See ONT reads mapping description in Materials and methods for more details.

*P*. *apterus* TAIMAN, a steroid receptor coactivator, is encoded in the linden bug by a single *tai* gene (Fig 1). The *tai* locus in our in-house assembled genome spans over 767 kbp (Fig 1A). *Taiman* transcripts were identified by mapping full-length Oxford Nanopore Technology transcriptomic reads to the *P*. *apterus taiman* genomic contig. Eight identified *tai* isoforms (Fig 1D) are generated by three mechanisms: (i) transcription from three alternative promoters, (ii) alternative read-through of exon 20, and (iii) alternative presence of exon 21. Each of the first three exons is transcribed from an independent promoter and is spliced to the canonical exon 4 (Fig 1A and 1D). Translation of six transcripts expressed from exons 1 and 2, *tai* iso-A-C, and iso-D-F, respectively, starts at the Methionine in exon 4, and those transcripts represent about 80% of *tai* expression. Transcripts expressed from exon 3 (*tai* iso-G, H) are prolonged by 40 amino acids (aa) at the N-termini, as a result of an alternative translational start site (Fig 1A and 1D). Transcripts *tai* iso-C, and iso-F represent minor isoforms in the brain. Both contain the read-through exon 20, and are translated into TAI-C protein isoform with extra 60 aa at the C-terminus but lack exons 21–24. Transcripts *tai* iso-A, iso-D, and iso-G include an alternative exon 21, which prolongs corresponding proteins by 45 aa, together constitute about 1/3 of *tai* brain expression.

#### *tai* silencing in *P*. *apterus* slows the circadian clock by 3–4 hours

**Fig 2:**
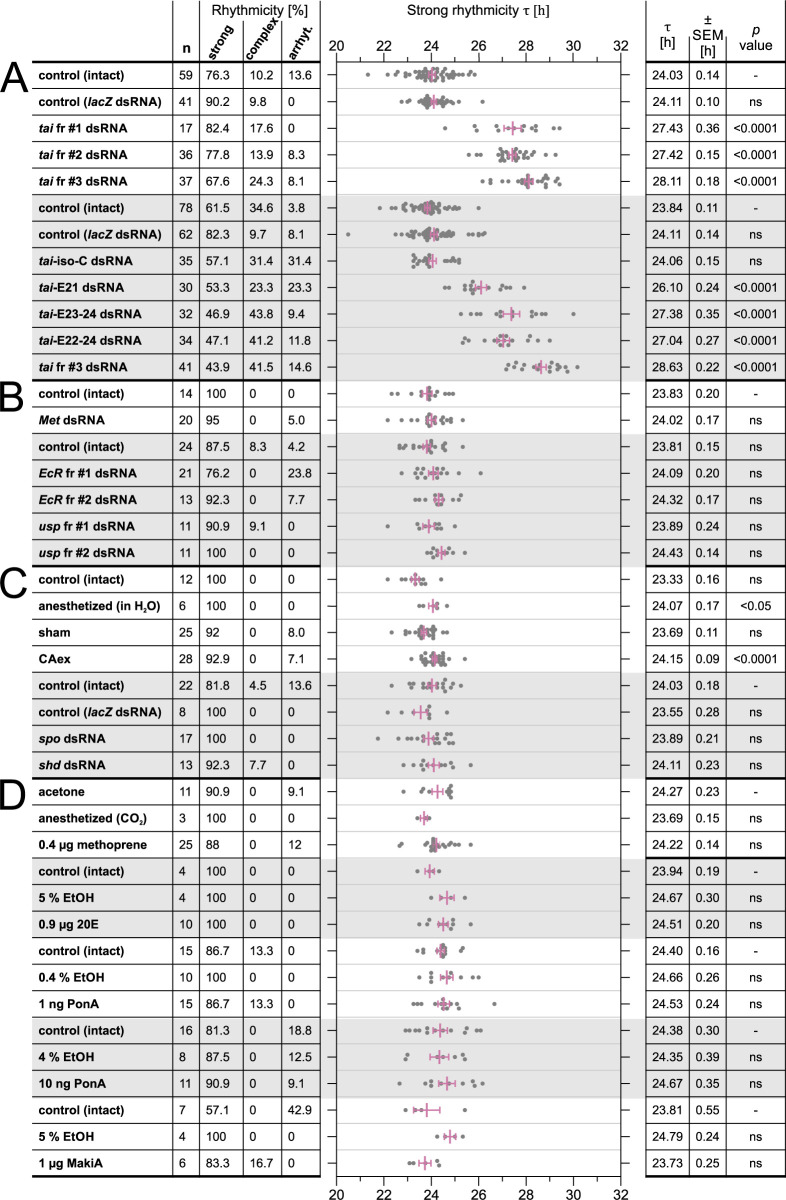
Knockdown of *taiman* slows down the pace of the circadian clock. *P*. *apterus* males were treated as indicated in the first column and their locomotor activity was measured in constant darkness at 25°C. The impact on behavioral rhythmicity is depicted as a percentage of males with strong rhythmicity, complex rhythmicity, and arrhythmicity. Individual free-running period (τ) values of males with strong rhythmicity (from column 3) are shown as individual dots, magenta lines represent the mean ± SEM (Standard Error of Mean). The mean τ, SEM, and statistical difference from the ‘control (intact)’ (*p* value) (One-way ANOVA with Dunnett’s multiple comparisons post hoc test for all analyses except for comparison of control (intact) and *Methoprene-tolerant* (*Met*) dsRNA, where unpaired two-tailed *t*-test was used). (A) Silencing of *taiman*, targeting all *tai* isoforms (white region) or *taiman* isoforms (grey region). (B) Silencing of juvenile hormone (JH) receptor *Met* (*t*(31) = 0.7152, *p* = 0.4799) (white region) and components of ecdysone receptor complex (*EcR* and *usp*) (grey region). (C) The effect of hormone production removal on the behavioral rhythmicity and τ. Juvenile hormone-producing *corpora allata* gland removal (CAex) (white region); knockdown of ecdysone-synthetizing enzymes encoded by *spook* and *shade* genes (grey region). (D) The effect of hormones or their mimics administration on *P*. *apterus* behavioral rhythmicity and τ. Topical JH mimic methoprene application (upper white region); injection: of 20-hydroxyecdysone (20E) (upper grey region), crustacean ponasterone A (middle white and lower grey regions), and natural ecdysteroid makisterone A (lower white region). Abbreviations and description: control (intact) = non-treated males, control (*lacZ* dsRNA) = *lacZ* double-stranded RNA fragment, *tai* = *taiman*, *tai*-iso-C = *taiman* isoform C, *tai*-E21 = *taiman* exon 21, fr #1, #2, and #3 represent non-overlapping dsRNA fragments, *Met* = *Methoprene*-*tolerant*, *EcR* = *Ecdysone receptor*, *usp* = *ultraspiracle*, anesthetized (in H_2_O) = anesthetized by submersing in water, sham = placebo surgery, CAex = surgical *corpora allata* removal, *spo* = *spook*, *shd* = *shade*, acetone = 4 μl of 100% acetone applied, anesthetized (CO_2_) = anesthetized by CO_2_ gas, methoprene = methoprene in acetone applied, 5% EtOH = 5% ethanol in H_2_O, 20E = 20-hydroxyecdysone in 5% EtOH, ponA = ponasterone A in EtOH, makiA = makisterone A in EtOH. n = number of measured males, rhythmicity: strong = percentage of males with one clear stable period, complex = rhythmicity with more components or unstable period, arrhythmic = no significant period, ns = statistically nonsignificant (p > .05).

**Fig 3:**
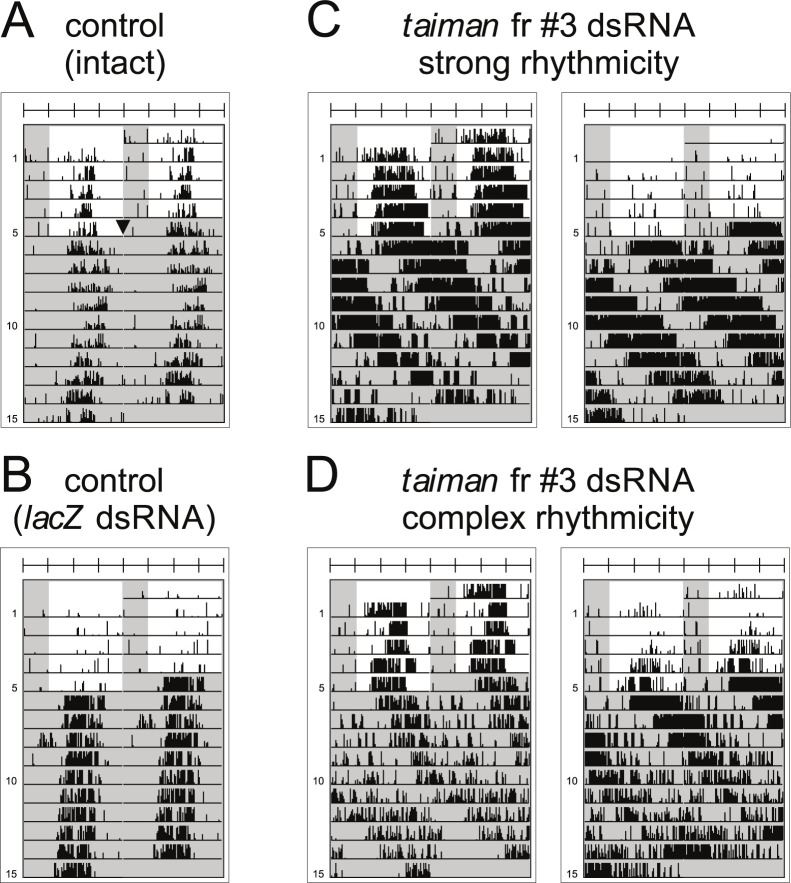
*P*. *apterus* locomotor activity after *taiman* knockdown presented as double-plotted actograms. The males were injected with *taiman* fr #3 dsRNA, loaded into locomotor activity monitors, and their activity was recorded at 25°C. Males were exposed to light-dark cycles for five days (depicted as white rectangles) and then were released to constant-dark conditions, indicated by a black arrowhead in actogram (A). Examples of (A) control (intact = wild-type), (B) control *lacZ* dsRNA injected, (C) males after *taiman* knockdown with strong rhythmicity (= one clear peak of locomotor activity), (D) males after *taiman* knockdown with complex rhythmicity (= more than one peak of the locomotor activity or free-running period has changed during the measurement). See materials and methods for a detailed description of strong and complex rhythmicity phenotype.

**Fig 4:**
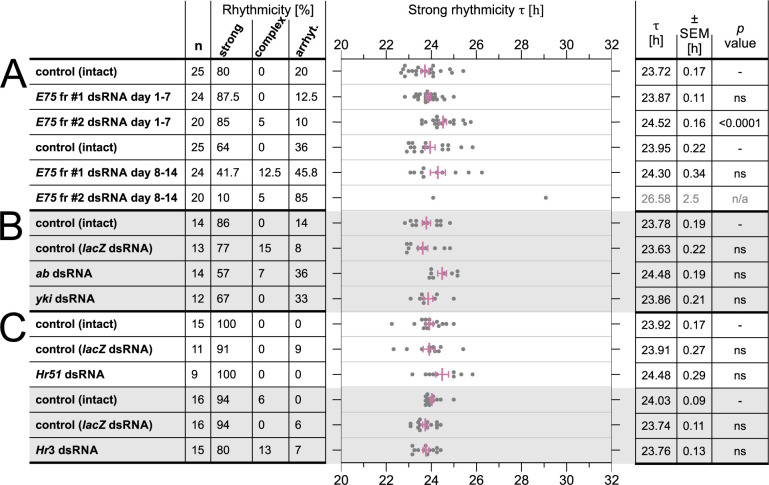
Knockdown of *E75*, *Hr51*, *Hr3*, *abrupt* and *yorkie*. *P*. *apterus* males were treated and analyzed as described in Fig 2. (A) The percentage of males with rhythmic, complex, or arrhythmic behavior after RNA interference against *P*. *apterus E75*. The behavior of the males was analyzed separately for the first 7 days in the dark (1–7 in DD, left panel), and for the next 7 days (8–14 in DD, right panel). The mean τ, SEM, and statistical difference from the ‘control (intact)’ (*p* value) were determined by One-way ANOVA with Dunnett’s multiple comparisons post hoc test (for days 1–7), and unpaired two-tailed *t*-test (t(24) = 0.9091, *p* = 0.3723) for intact vs. *E75* fr #2. (B-C) *P*. *apterus* males were treated as indicated in the first row and their locomotor activity was measured and analyzed as described in Fig 2 (standard 10 days in DD). The mean τ, SEM, and statistical difference from the ‘control (intact)’ (*p* value) (One-way ANOVA with Dunnett’s multiple comparisons post hoc test). ns = statistically nonsignificant (p > .05), n/a = not applicable.

The knockdown of *tai* via RNA interference (RNAi) dramatically slowed the circadian clock FRP (= τ, tau) to 27.42–28.63 hours (Figs 2A and 3). τ was evaluated only in males, because contrary to females, their assay performance is not affected by reproduction-related phenomena. All three tested *tai* non-overlapping dsRNA fragments, targeting all detected isoforms, significantly slowed the pace of the clock (Figs 2A and 3C), but the ratio of perfectly rhythmic linden bug males was comparable to control (intact) males (Fig 2A). The rhythmicity of control (intact) males was in some cases lower than in males with treatment (i.e., strong rhythmicity % of “control (intact)” in Figs 2A and 4A), most likely due to heterogeneity in the population of cohort used in particular experiment. The ratio of males with more components or unstable period (= complex rhythmicity) was slightly higher in *tai* RNAi males (Figs 2A and 3D). The use of three independent *tai* dsRNA fragments, consonant in the circadian phenotype, strongly supports *tai* RNAi specificity and excludes the possibility of *tai* RNAi phenotype being caused by an off-target effect.

We decided to test whether TAI, a heterodimerizing partner of MET in a JH receptor complex, could execute its circadian clock modulatory function through MET/JH signaling pathway. However, the knockdown of *Met* had no effect on the rhythmicity or τ (Fig 2B). To further test the requirement of JH for the circadian clock, we surgically removed *corpora allata*, the endogenous source of JH (allatectomy, CAex) in males 24–48 hours after adult ecdysis that were reared under the long-day (18:6 h light/dark) regime, which is the photoperiod stimulating activity of CA. The rhythmicity of CAex males was fully comparable to intact, sham-operated, and *lacZ* dsRNA-injected control males (Fig 2C). Although allatectomized males’ τ of 24.15 hours is close to the expected 24-h period, it was significantly different from 23.33 hours measured in control (intact) males. The mean τ of males anesthetized by submersion in water was 24.07 hours, being almost identical to CAex-treated males. The period in sham-operated males was 23.69 hours and close to control males in other experiments (Fig 2C). Since the allatectomy had no effect on rhythmicity and τ, we decided to perform an inverse experiment, in which we administered JH mimic methoprene and its vehicle, acetone. In both cases, application on adult males induced no change in the rhythmicity or τ (Fig 2D), suggesting little or no role of JH in the linden bug circadian clock.

Thus, insensitivity of the linden bug circadian clock to disrupted JH signaling, contrasting to the strong *tai* knockdown effect, suggested a clock-specific (JH-independent) mechanism for TAI functions. One of the possible mechanisms could be TAI coactivating role in ecdysone signaling, in which TAI binding to EcR/USP complex permits target gene expression [25].

### *P*. *apterus* circadian clock is ecdysone signaling-independent

We decided to comprehensively test the involvement of ecdysone signaling in the *P*. *apterus* circadian clock by (i) determination of ecdysone levels, (ii) disrupting endogenous ecdysone synthesis, (iii) removing intracellular ecdysone receptor, and (iv) the application of exogenous ecdysteroids and ecdysone mimics.

Makisterone A (makiA) is a major ecdysteroid of some true bugs (Heteroptera, Pentatomomorpha), including the linden bug *P*. *apterus* [40,41] (S1 Fig). MakiA relative amount determined by liquid chromatography is high in seven-day-old fifth-instar *lacZ* dsRNA-injected larvae and the freshly eclosed *P*. *apterus* males. The amount drops to about 10% in seven-day-old males (S1 Fig). We decided to block ecdysone synthesis by knocking down two crucial cytochrome P_450_ enzymes, encoded by *spook* (*spo*, CYP307a1) and *shade* (*shd*, CYP314a1) genes and ecdysone intracellular receptor by knocking down *EcR* and *usp* genes. Linden bug *spo*-, *shd*-, *EcR-* and *usp*-silenced males were fully rhythmic and their τ was unchanged when compared to the control intact and *lacZ* dsRNA injected males (Fig 2B and 2C).

**Fig 5:**
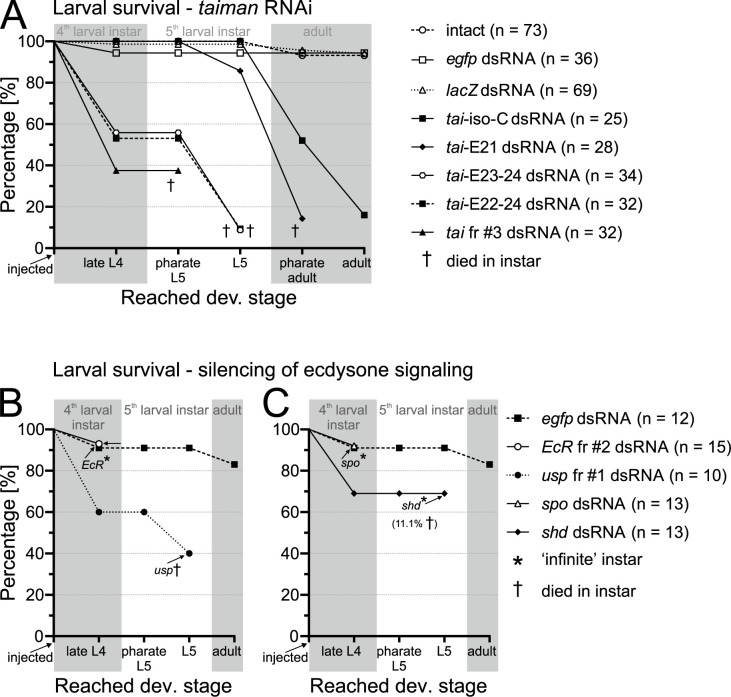
Knockdown of *P*. *apterus taiman* blocks larval development. Freshly hatched *P*. *apterus* 4^th^ instar larvae were injected with dsRNA or left untreated (intact). (A) Isoform-specific knockdown was more pronounced the more *taiman* isoforms were targeted, see Fig 1 for details of which isoforms were targeted by given dsRNA fragments. Animals marked with † died within 2–3 days after reaching the depicted developmental stage. Data presented in (B) and (C) were generated in the same experiment but plotted separately for higher clarity. Data of *egfp* dsRNA-injected control animals are identical in both (B) and (C) plots. Animals marked with * stopped developing, stayed in the instar for up to one month, and ultimately died as L4 (*EcR*, *spo*) or L5 (*shd*) larvae.

Since blocked ecdysone synthesis or ecdysone binding by the receptor had no effect on circadian rhythms, we tested the RNAi efficacy in larval development, where ecdysone signaling plays a crucial role. Firstly, injection of *spo* dsRNA into freshly ecdysed fifth-instar (= L5) larvae dropped makiA levels to about 4% of the amount detected in control larvae (S1 Fig), proving *spo* RNAi to be an effective way of makiA level reduction. *EcR*, *spo*, *shd*, and to a lesser degree also *usp* dsRNA, injected into the one-day-old 4^th^ instar larvae (= L4) blocked ecdysis (Fig 5B and 5C), as expected for genes essential for ecdysone signaling. *EcR* and *spo* RNAi larvae never molted to the 5^th^ instar, stayed in the ‘infinite’ 4^th^ instar for up to one month, and eventually died. The majority (91.7%) of control *egfp* dsRNA-injected larvae molted to L5 instar within 4–5 days and 90.9% of them developed into adults. Interestingly, 60% of *shd* and 40% of *usp* dsRNA injected L4 larvae molted to the 5^th^ instar, but *usp* died shortly after the ecdysis, and 88.9% of *shd* RNAi (L5) larvae became ‘infinite’ L5 larvae. Taken together, larval RNAi of (mainly) *EcR* and *spo* proved to be an efficient way to downregulate ecdysone signaling in the linden bug.

In the inverse experiment, injection of the linden bug major ecdysteroid makisterone A, insect most common 20-hydroxyecdysone (20E), or ecdysone mimic ponasterone A into *P*. *apterus* adult males (at Zeitgeber Time = 3–5), affected neither their rhythmicity nor τ, and the phenotypes were comparable to intact, *lacZ* dsRNA-injected, 0.4% EtOH- and 5% EtOH-treated control males (Fig 2D).

The ecdysone signaling pathway regulates expression of many target genes, including nuclear receptors (NR) [23]. We knocked down three NR linked with reported function in *Drosophila* and *Thermobia* circadian clock, *Hr51*, *Hr3*, and *E75*. *Hr51*- and *Hr3*-silenced linden bug males were rhythmic and their τ was not significantly changed (Fig 4C). Interestingly, *E75*-silenced males were rhythmic between the 1^st^ and 7^th^ day in constant darkness but became gradually arrhythmic in the subsequent seven days (Fig 4A). The effect was more prominent in *E75* RNAi fragment #2, where 85% of males became arrhythmic. τ determined in the rhythmic males from *E75* RNAi fr #2 measurement was significantly longer (24.52 hours) during the first 7 days in darkness when compared to control (intact) males (Fig 4A).

Ecdysone signaling in the fruit fly is negatively regulated by the Zinc finger C2H2-type transcription factor ABRUPT (AB), which attenuates ecdysone signaling by binding the bHLH domain of TAI by its BTB (Broad-Complex, Tramtrack and Bric a brac) domain [42]. Although no role for AB in *Drosophila* circadian rhythm is known and its expression was reported from a subset of dorsal lateral neurons (LNd) rather than from ventral lateral neurons (LNv) expressing EcR, HR51 (= DHR51), and E75B [30,34,43], we decided to test *ab* RNAi effect on the linden bug circadian behavior. Contrary to its function as a negative regulator of TAI in ecdysone signaling, τ of *ab*-silenced linden bug males was not shorter than the τ in control (intact) males, although the rhythmicity of *ab* RNAi males was slightly lower (Fig 4B).

Similarly, TAI facilitates physical interaction between a WW (tryptophan-tryptophan) domain of YORKIE (YKI), a transcriptional co-activator of the Hippo signaling pathway, and EcR, to regulate *Drosophila* imaginal disc growth [44]. We silenced *P*. *apterus yki* via RNAi to test its possible effect on the linden bug clock pace, but the τ was comparable to the free-running period in the control (intact) and *lacZ* dsRNA-injected males (Fig 4B).

Taken together, out of all our ecdysone signaling manipulations, only *taiman* silencing had a strong effect on the pace of the linden bug circadian clock, corroborating JH- and ecdysone-independent function of TAI in the linden bug circadian clock.

### *Taiman* circadian phenotype complies with the number of *tai* isoforms silenced

The role of TAIMAN in the modulation of the *P*. *apterus* circadian clock seems to be JH- and ecdysone-independent. The contrast between striking *tai* RNAi circadian phenotype and no effect of JH and ecdysone signaling manipulations on males’ free-running period or rhythmicity encouraged us to further support the function of TAI in the linden bug physiology and the circadian clock. We decided to perform isoform-specific *tai* knockdowns and to test whether TAI clock modulatory function can be associated with specific *tai* isoform(s). Eight *tai* isoforms were detected at the level of mRNA, which can be translated into five TAI proteins in *P*. *apterus* (Fig 1D).

### Additive effect of isoform-specific *taiman* silencing on the clock

We designed and cloned *tai* fragments targeting (i) isoform-specific exon 20 extension (*tai*-iso-C RNAi), silencing a single (protein) isoform TAI-C, (ii) alternative exon 21 (*tai*-E21 RNAi), silencing TAI-A and TAI-D, (iii) exons E23-24 (*tai*-E23-24 RNAi) or E22-24 (*tai*-E22-24 RNAi), silencing TAI-A, TAI-B, TAI-D, and TAI-E isoforms (Fig 1C). Common *tai* RNAi fragment #3, silencing all isoforms, was used as a positive control. The experimental design was the same as presented for common *tai* RNAi fr #1, #2, and #3 (Figs 1 and 2A). Although the rhythmicity of all males measured in the *taiman* isoform-specific RNAi experiment was lower, including control intact and *lacZ* dsRNA-injected males, the ratio of strongly rhythmic males gradually decreased with the number of silenced isoforms and dropped to 43.9% in *tai* fr #3 RNAi.

Removal of TAI-C isoform via *tai*-iso-C RNAi had no effect on τ and was comparable to intact and *lacZ* dsRNA injected control males (Fig 2A). Knockdown of *tai* isoforms containing alternative exon 21 prolonged τ to 26.10 hrs. Silencing performed by *tai*-E23-24 and *tai*-E22-24 RNAi yielded τ of 27.38 and 27.04, respectively, which is close to τ for common *tai* fr #1 and #2 RNAi (Fig 2A). *tai* fr #3 RNAi, used as a positive control, slowed τ to 28.63 hrs. Based on all these results, the effect of *tai* isoform depletion on τ seems to be rather additive than dependent on specific isoform knockdown.

### *taiman* isoforms in development and reproduction

We decided to apply isoform-specific *tai* RNAi in other TAI-govern processes well-established in insects: development and reproduction. Over 93% of intact or *egfp*- and *lacZ*-dsRNA-injected larvae developed into adults (Fig 5A). In larvae injected with dsRNA fragments targeting the most, *tai*-E23-24 and *tai*-E22-24, and all, *tai* fr #3, isoforms died no later than in the 5^th^ instar, with 44.1%, 46.9%, and 62.5% of them dying as late 4^th^ instar larvae, and 8.8%, 9.4%, and 0%, respectively, reaching late L5 instar (Fig 5A). Knockdown of isoforms containing exon 21 (*tai*-E21) permitted reaching late L5 in 85.7% of injected larvae, but only 14.3% of total larvae entered L5/adult ecdysis and died as pharate adults. All larvae injected with *tai*-iso-C dsRNA developed normally up to the late L5 instar, 52% of them molted to the pharate adult stage and only 16% developed into adults. All *tai* RNAi fragments tested had a developmental phenotype but differed in the reached developmental stage and penetrance level.

**Fig 6:**
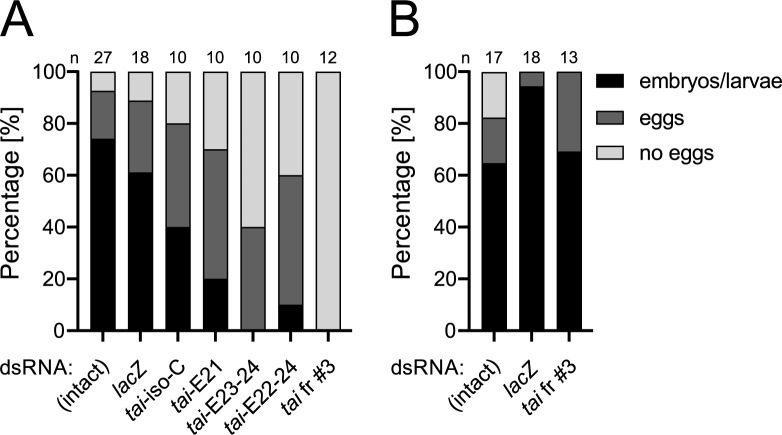
Silencing of *taiman* blocks egg development in *P*. *apterus* females. (A) Two-day-old *P*. *apterus* adult females were injected with depicted dsRNA or left untreated, and upon addition of males two days later, were scored for egg-laying and embryonal/larval development. The majority of control females, 92.6% of intact and 88.9% of *lacZ* dsRNA injected females, laid eggs, whereas the ratio of egg-laying females dropped to 40%, 60%, and 0% in *tai*-E23-24, *tai*-E22-24 and *tai* fr #3 dsRNA injected females, respectively. (B) No effect of *tai* silencing was observed on male fecundity, as two-day-old *P*. *apterus* males, injected with *tai* fr #3 dsRNA, control *lacZ* dsRNA, or left untreated, were able to fertilize *P*. *apterus* females.

The effect of isoform-specific *tai* knockdown on *P*. *apterus* reproduction was tested by injection of dsRNA in two-day-old virgin females, crossed with males two days later, and scored for an egg laying and presence of embryos/larvae (Fig 6A). Knockdown of all *tai* isoforms, performed by *tai* fr #3 RNAi, led to complete female sterility. Depletion of most *tai* isoforms by *tai*-E23-24 and *tai*-E22-24 RNAi allowed only 40% and 60% of females, respectively, to lay eggs. Furthermore, embryonic development was blocked in most eggs that were laid by *tai*-E23-24 and *tai*-E22-24 dsRNA-treated females. Females injected with *tai*-iso-C and *tai*-E21 dsRNA showed only a slight decrease in egg-laying and development of embryos and larvae compared to control intact and *lacZ* dsRNA-injected females. Interestingly, females crossed with two-day-old *tai* fr #3 RNAi males (= paternal RNAi) laid eggs at a level comparable to females crossed with control intact and *lacZ* dsRNA-injected males (Fig 6B). Data suggest that paternal *tai* RNAi has no effect on female egg laying and embryonic/larval development. Contrary to the proposed role of *tai* isoforms in development, isoform-specific *tai* RNAi experiments do not support a specific role for any *tai* isoforms in circadian clock.

### TAIMAN clock modulatory function is conserved in the cockroach *Blattella germanica*

TAI seems to be a robust modulator of the linden bug circadian clock. Whether TAI clock modulatory function is a common feature in other insects is unknown. Therefore, we decided to address *taiman* role in the circadian clock of the German cockroach *Blattella germanica* (Blattodea), an insect species that separated from true bugs (Heteroptera) ~ 380 mya [45].

**Fig 7:**
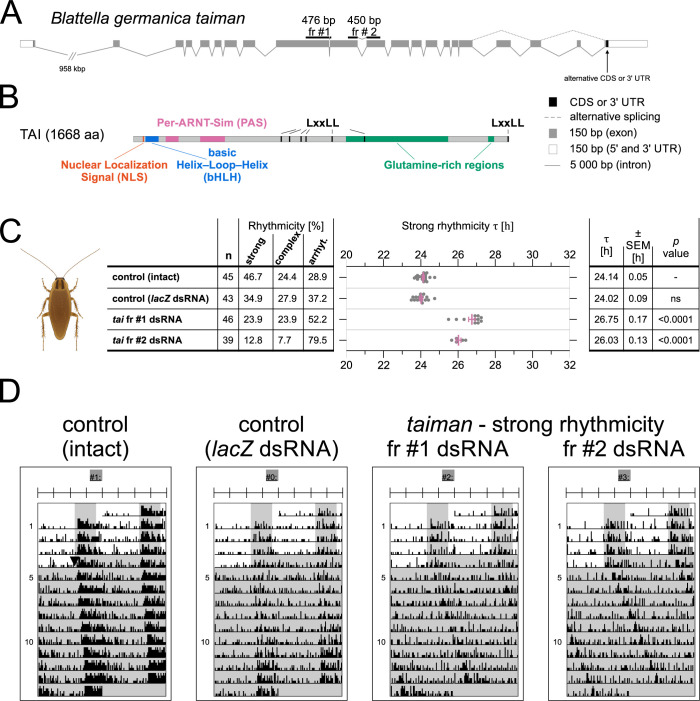
*taiman* knockdown in cockroach *Blattella germanica* slows down the pace of the circadian clock. (A) *B*. *germanica taiman* gene locus with mapped transcripts from [46]. Alternatively spliced exons and the position of RNA interference fragments are depicted. Note the different scales of exons and introns. (B) The domain structure of *B*. *germanica* TAIMAN protein, represented by (longest) TAI isoform (1668 amino acids, encoded by HG965205.1 transcript). See Fig 1 for the domain description. (C) *B*. *germanica* males were injected with 1 μl of *tai* fragment #1 or #2 dsRNA, or control *lacZ* dsRNA, or left without treatment (control intact), and individually placed in Petri dishes partially filled with agar, providing food and water. Males were entrained for 4 days (16 h light/8 h dark) and then their locomotor activity was measured in constant darkness at 27°C. The impact on behavioral rhythmicity is depicted as a percentage of males with strong rhythmicity, complex rhythmicity, and arrhythmicity. Individual free-running period (τ) values of males with strong rhythmicity (from column 3) are shown as individual dots, magenta lines represent the mean ± SEM (Standard Error of Mean). The mean τ, SEM, and statistical difference from the ‘control (intact)’ (*p* value) (One-way ANOVA with Dunnett’s multiple comparisons post hoc test). ns = statistically nonsignificant (p > .05). See details on FRP determination in Materials and methods. (D) Examples of *B*. *germanica* locomotor activity double-plotted actograms of males with strong rhythmicity from (C). The males were either injected with dsRNA (*lacZ* or *tai*) or left untreated (control intact males). Males were exposed to light-dark cycles for four days (depicted as white rectangles) and then were released to constant-dark conditions, indicated by a black arrowhead in the control (intact) actogram. Notice predominant nocturnal locomotor activity in all treatments. See Materials and methods for a detailed description of strong and complex rhythmicity phenotype.

*B*. *germanica* possesses a single *tai* gene, which we reconstructed from published genomic contigs and transcripts (Fig 7A) [46; see methods for more details]. Our analysis suggests four published *B*. *germanica tai* coding sequences are longer at their 5’end compared to the annotated gene model in (PYGN01000796.1) and encode an extra 30 amino acids at the N terminal end. Predicted *B*. *germanica* TAI is very similar to other insect TAI proteins and contains all conserved domains and motifs (Fig 7B).

For functional analysis we designed two non-overlapping dsRNA fragments, which target all known *B*. *germanica tai* transcripts (Fig 7A). *B*. *germanica* males were injected with 1 μl of *tai* fragment #1 or #2 dsRNA, or control *lacZ* dsRNA, or left without treatment (control intact). After a 4-day entrainment (16 h light/8 h dark), the locomotor activity was measured in constant darkness at 27°C. The portion of rhythmic males was low in the whole experiment, ranging from 46.7% in control intact males to 23.9% and 12.8% in *tai* dsRNA fr #1 and #2 injected males (Fig 7C), respectively. However, *tai* silenced males with strong rhythmicity had τ 26.8 and 26.03 hours (Fig 7C and 7D). The slow pace of the clock in *B*. *germanica* and *P*. *apterus* males thus seems to be a shared feature of at least two distant insect species.

## Discussion

Here, we demonstrated *taiman* as a new modulator of the insect circadian clock. TAIMAN is the only insect ortholog of mammalian Steroid Receptor Coactivators [25] and represents a pleiotropic protein engaged in several signaling pathways, out of which juvenile hormone and ecdysone signaling pathways are the most studied [22,25,37,42,47]. TAI seems to be a robust modulator of insect locomotor circadian rhythms as all *tai* common dsRNA fragments lengthened the free-running period in at least two insect species, the linden bug *P*. *apterus* and German cockroach *B*. *germanica* (Figs 2A and 7C). Longer τ was also measured upon *P*. *apterus tai* isoform-specific knockdowns, except for *tai*-iso-C dsRNA targeting rare isoforms (Fig 1D), which left τ unchanged (Fig 2A). Mouse TAI ortholog Steroid Receptor Coactivator-2 has been shown as a transcriptional coactivator of BMAL1/CLOCK [8]. Although the average τ of SRC-2^-/-^ mutant mice was not changed compared to controls, τ varied among individual mice. Moreover, mutant SRC-2^-/-^ mice expressed an abnormal behavioral pattern in locomotor wheel-running behavior [8]. Thus, the TAI/SRC-2 clock modulatory role in two insect species and mice might represent a conservative feature of the circadian clock.

TAI is indispensable for the JH signaling pathway, and *tai* silencing blocks larval development [22,46,48] and reproduction [22,49]. The function of JH in the insect circadian clock stays elusive. The removal of *corpora allata* (allatectomy) in the linden bug males had no effect on the pace of the clock (this work), a phenotype found also in the honeybee (*Apis mellifera*) [50] and bumble bee (*Bombus terrestris*) [51]. The same circadian clock JH-insensitivity was found upon *Met* knockdown and when JH mimic methoprene was administered to the linden bug males [this work]. The fact that JH level manipulations are not reflected in the clock pace does not mean that either crosstalk between clock proteins and JH receptor proteins MET and TAI or JH-driven clock genes’ expression is not possible. These interactions are often localized in peripheral tissues, such as in the linden bug *P*. *apterus* fat body [38] and gut [39], and the mosquito *Aedes aegypti* fat body [37], and thus suggest a pleiotropic function of the clock proteins rather than direct involvement of the JH signaling in the control of the circadian rhythms. A notable similarity in tissue-specific clock architecture is found in mammals, where the importance of individual TTFL differs in a tissue-specific manner, which accounts for organ-specific clock gene expression and contributes to the hierarchical organization of the clock [52].

Ecdysone signaling was linked with circadian rhythms in *Drosophila* [30,36] but the involvement of TAI in the *Drosophila* circadian clock has not been reported. The ecdysone signaling role in *Drosophila* contrasts with our findings in the linden bug males, where *EcR* and *usp* knockdown had no effect either on rhythmicity or τ.

In *D*. *melanogaster*, EcR overexpression, knockdown, or expression of a dominant-negative EcR form in clock neurons caused higher arrhythmicity, weakened rhythm strength, and in some driver-UAS construct combinations lengthened τ [30,36]. On the other hand, *Drosophila EcR* (and *usp*) silencing using *miRNA* overexpression in PDF-positive neurons had a minimal effect on locomotor activity rhythmicity and no effect on τ [34], showing how delicate those experiments are. The linden bug *EcR* and *usp* dsRNA were injected into 1-2-day-old adult males, avoiding the detrimental effect of *EcR* knockdown throughout the development, as described for the *Drosophila* small ventral lateral clock neurons (s-LNvs) [53]. Moreover, the knockdown of two ecdysone-inducible nuclear receptors, E75B and HR51, in *Drosophila* s-LNvs, causes weaker circadian behavior or arrhythmicity [30,34,54,55]. Although *P*. *apterus* males are rhythmic after *Hr51* and *Hr3* knockdown and only the *Hr51* free-running period is slightly (not significantly) longer, *E75* silenced males gradually became arrhythmic and the rhythmic males had slightly longer τ, although only with one RNAi fragment. The onset of arrhythmicity after *E75* dsRNA injection was detected at 8–14 days in DD (13–19 days after dsRNA injection) (Fig 4A), contrasting with strong circadian phenotypes published for core circadian genes [10]. In *Drosophila*, E75B physically interacts with PER and represses *Clk* expression in insect and mammalian cell cultures, and E75B was postulated as a factor protecting circadian rhythms from stress [30]. Whether *P*. *apterus* E75 interacts with the linden bug PER is unknown, but 43.3–50% of *P*. *apterus per* RNAi and 15–38% *per* null mutant males were still rhythmic with τ ~ 19–21 hours [10], suggesting lower dependence of the linden bug clock on PER. Interestingly, *Drosophila E75* is not only a 20E-inducible early gene but can be induced by JH III (or JH mimic methoprene) in *Drosophila* S2 cells in the absence of 20E [56].

Ecdysone titer following daily rhythms was determined in several insect species [26–29] and we thus decided to test the effect of makisterone A (makiA), in insects the most common 20-hydroxyecdysone (20E), and ecdysone-mimic ponasterone A administration on *P*. *apterus* males’ rhythmicity and τ (Fig 2D). None of the aforementioned compounds changed the linden bug rhythmicity or τ and agreed with the results obtained after *EcR* and *usp* knockdowns. Ecdysone signaling-independent behavioral locomotor activity in linden bug adult males (Fig 2B and 2D) contrasts with the effect of disrupted ecdysone signaling through the bug larval development (Fig 5B and 5C). Although the relative makiA level was not determined in fourth-instar larvae, the amount of makiA in (late) fifth-instar larvae is comparable to levels detected in freshly hatched adult males and the titer gradually decreases throughout the first week of males’ adulthood. The relative amount of makiA in fifth-instar larvae can be effectively decreased by silencing *spo* (S1 Fig), thus proving the efficacy of *spo* knockdown. The larval and adult RNAi in *P*. *apterus* is equally efficient (compare *Met* RNAi in [19,22,57]). Larval *spo*, *shd*, *EcR*, and *usp* knockdown in *P*. *apterus* elicited strong developmental phenotype (Fig 5B and 5C), yet silencing of the same genes in adult males affected neither the rhythmicity nor τ, further supporting the linden bug ecdysone-independent clock rhythms (Fig 2C).

**Fig 8:**
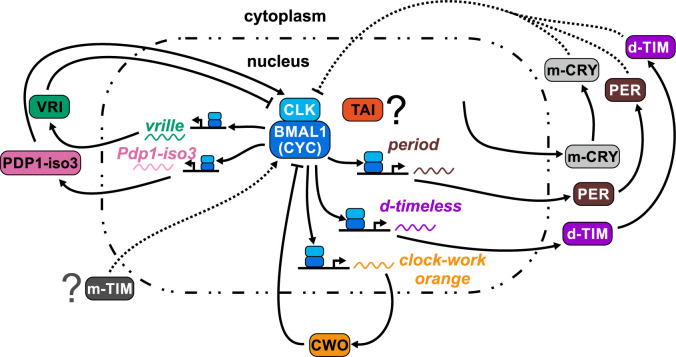
The scheme of the circadian clock in *P*. *apterus*. The scheme is based on experimental data published by [10]. The position of TAIMAN protein and the mechanism of action are unclear.

The mechanism through which TAI modulates linden bug circadian rhythms is unclear (Fig 8). TAI mammalian ortholog SRC-2 binds BMAL1 through LxxLL protein motifs and pulls down both BMAL1 and CLK [8]. *Clk* and *cyc* knockdown in *P*. *apterus* resulted in a complete arrhythmicity [10]. LxxLL motifs are known to interface TAI/SRC interaction with nuclear receptors including EcR and USP in insects [25,58]. BMAL1/CLK/SRC-2 drives expression of PER1 by binding to *per1* promoter but SRC-2/BMAL1 binding was also enriched in several other known circadian genes, together with genes involved in metabolism [8]. *P*. *apterus* and *B*. *germanica* TAI-depleted males have a free-running period longer by several hours, suggesting TAI ‘speeds up’ the clock pace in control, non-treated males.

It will be interesting to see whether TAI participates in the *Drosophila* circadian clock. Although the circadian clock is generally well conserved in insects [59,60], there are several noteworthy unique features of *Drosophila* clock machinery: For example, the transactivation domain (TAD) is localized only to CLK in the fruit fly, whereas CYC does not have this domain. The same pattern is found only in Cyclorrhapha (a subgroup of Diptera), whereas the TAD is localized to CYC/BMAL1 in the other bilaterians (Bilateria, most animals except sponges, placozoans, cnidarians, and ctenophores). TAD-dependent repression of BMAL1 requires m-CRY [61], a protein that has been lost in *Drosophila*, and the transition of TAD from BMAL1 to CLK corresponds exactly to the loss of m-CRY [62]. In mice, SRC-2 interacts with BMAL1-CLK and coregulates their function [8]. At this point, it is unclear whether TAI physically interacts with BMAL1 or CLK in *P*. *apterus* and *B*. *germanica*. Given the above-described modifications of CLK-CYC in *Drosophila*, it will be interesting to see whether TAI participates in the *Drosophila* (cyclorrhaphan) clock, and if so, whether the role is similar to that of *P*. *apterus* and *B*. *germanica*, or whether some *Drosophila*-specific modifications are identified.

**Fig 9:**
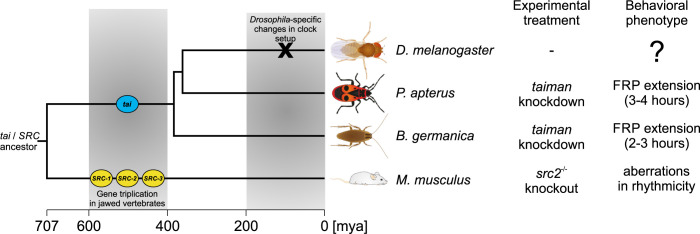
Graphical summary of TAIMAN/Steroid Receptor Coactivator 2 role in circadian clock. *tai*/*SRC* ancestor gene got triplicated in a jawed vertebrate lineage, whereas insects possess a single *tai* gene. Although *Pyrrhocoris apterus* and *Blattella germanica* lineages separated ~380 million years ago, *tai* silencing in both species slowed the pace of the circadian clock. Homozygous *Steroid Receptor Coactivator-2* (*SRC-2*) knockout (*SRC-2*^-/-^) mice (*Mus musculus*) exhibit a range of aberrant behavioral activities during light/dark cycles, from bimodal/phase-advanced wheel-running locomotor activity to complete arrhythmicity [8]. Testing TAI involvement in the fruit fly *Drosophila melanogaster* circadian clock would be of enormous interest, as the *D*. *melanogaster* clock setup differs in several aspects. See text for more details.

This presented study introduces steroid receptor coactivator TAIMAN as a new insect circadian clock factor. A similar role of insect TAIMAN and SRC-2 in mammals suggests that TAI/SRC clock function has been conserved for more than 500 million years (Fig 9). Although our results suggest that TAIMAN function in the insect circadian clock is ecdysone- and juvenile hormone-independent, the exact mechanism through which TAIMAN modulates the circadian clock function remains elusive.

## Materials and methods

### *Animal* rearing

#### *Pyrrhocoris apterus* rearing

Bugs (short-winged form) were maintained in 0.5-liter jars at 25°C and were supplemented with dry linden seeds (*Tilia cordata*) and water *ad libitum*. Animals were kept at a reproduction-permitting photoperiod of 18 h light (500–1000 lux) and 6 h dark. Adult males of the linden bug strain ‘Roana’ were used for locomotor activity measurements. Larvae of strain ‘Oldrichovec’ [63] were used for developmental experiments; fifth-instar larvae and adult males were used for makisterone A relative level determination.

#### *Blattella germanica* rearing

Roaches were kept in buckets covered with meshed lids, with a layer of sawdust at the bottom at a temperature of 23–25°C, supplemented with dried bakery products and crushed cat food pellets and water *ad libitum*, under a 16 h light and 8 h dark photoperiod.

### Gene identification, cloning, RNA interference

*Drosophila* protein homologs of studied genes were downloaded from GenBank or FlyBase databases and searched in the in-house *Pyrrhocoris apterus* transcriptomic database using BLAST (Basic Local Alignment Search Tool) algorithm (tblastn) in Geneious Prime 2022 (Biomatters, Auckland, New Zealand). Identified sequences were blasted against insects’ proteins in GenBank to verify their identity. Published *Blattella germanica taiman* transcript sequences [46], mapped to genomic contigs (see ‘*taiman* gene models’ section) were used for cloning primer design. Cloning primers (Table 1) were used to amplify gene fragments from various *P*. *apterus* or *B*. *germanica* cDNA libraries by PCR using PP MasterMix (Top-Bio) and cloned to pGEM-T easy vector (Promega). *P*. *apterus tai*-iso-C forward primer CGCCGAACCAACAGTTACCA binds a common *tai* region, upstream to the *tai*-C and *tai*-F isoform-specific coding region. Thus, PCR product resulting from *tai*-iso-C forward and reverse primers amplification was digested by SexAI restriction enzyme, removing the common *tai* region sequence, and the digested fragment was then cloned to pGEM-T easy vector. Fragment of *ß*-*galactosidase* gene (*lacZ*) was used as a negative control. 720-bp-long *egfp* ORF fragment in pEGFP-N1 (Clontech) was used as a negative control for larval developmental experiments. pEGFP-N1 was digested with SalI and NotI restriction enzymes and subcloned to pBlueScript KS (-) plasmid. T3 promoter in pBlueScript plasmid was replaced by T7 promoter in in vitro dsRNA transcription template by using M13F 5’-GTAAAACGACGGCCAGT-3’ and Blue-RNAi-R 5’-TAATACGACTCACTATAGGGAACAAAAG-3’ primers (T7 promoter underlined). All cloned gene fragments were verified by Sanger sequencing. SP6 promoter in pGEM-T easy vector was replaced for T7 promoter by PCR amplification using M13F and 5’- TAATACGACTCACTATAGGGGACACTATAGAATACT-3’ primers (T7 promoter underlined) and the purified PCR product was used for double-stranded RNA synthesis using T7 MEGAscript RNAi Kit (Ambion/Invitrogen) according to the manufacturer’s manual.

**Table 1:** Primer sets for cloning of cDNA fragments for dsRNA synthesis.

	Forward primer (sequence 5’-3’)	Reverse primer (sequence 5’-3’)
*EcR* fr #1	TGAGGAAGATTTGAGACGGATTAG	CTGAAGGTCGCTCTGAGAATATC
*EcR* fr #2	CTGTGATGGCTCGAAGAAGAA	AGGTACTACCCTACCAAGAGTG
*usp* fr #1	GGGCAGAGGTTTGAGTTTAGAG	TAGTTCTTTGCCTCTCCTCCT
*usp* fr #2	GTTGAAAGCACGAGCAGTTT	CTCAGTCGTGTATGCTCTTCC
*tai* fr #1	CATGAGCAGTCTGTCAGTGA	TCTTCGAGATTCTTGTGCGC
*tai* fr #2	ACGAAGAAAACAATCAGGATAA	CAGGGGAATCAGTGGTAA
*tai* fr #3	CAATCTGTCCAACAACAACA	CACCACCACTTGTGTAATTT
*tai*-iso-C†	CGCCGAACCAACAGTTACCA	GGATTATTTTGATTAGGAGGA
*tai*-E21	TGGTACTAGGGTGGGTTA	GTGGGGATGGTGTAGTAA
*tai*-E23-24	*AGTGAGTGATACCTCAAAAA	*ATTATTCCGAAAGAAGC
*tai*-E22-24	GGTGGTAATAACGGGGTT	CGATCTTGACACTTCTTCC
*Met*	ATGGTATCCTCATCTCCTAAG	GTGTGTTGATGCAGATGAATG
*E75* fr #1	GGTGACAAGGCTTCTGGATT	GCTGTGTATCGAGTCCTTCTTC
*E75* fr #2	ACGCACTTAAGTTGACAGACTC	CTAGTTTCCTGAACCTGTGTGG
*shade*	TCACGAGGCGCACGCAGATTTGTA	GGATAACTCGCTCGCCGTCTCGTC
*spook*	CCGCACTGGACCCGATAAAA	TGAGCCAGGGCATAAAGTCT
*Hr3*	AGAAGTCAGTCGTCGGTGG	TACTCGGTCTGGGGTCGTAG
*Hr51*	ACTACAGCAGCCAAACAG	TAGACCAAGTCCCACAGG
*abrupt*	ATGGGTGGGCTTGGTGGTA	TTTCTGTTGTTCCTTTGTTGGG
*yorkie*	CCTTCCACATCGCCACAACCACA	GGCCTAAACCAGAATCCGCAGACTC
*Bg tai* fr #1	ACCACACCTGCTAATCCGT	TCGTCATCGCTCTTTTCGTTC
*Bg tai* fr #2	CATCAGGCACCACACAACC	TATCCGACGCACCAACCAA

* T7 promotor sequence TAATACGACTCACTATAGGG was used as an extension of the primer

† see details of cloning in the section Gene identification and cloning

Coding sequences of *P*. *apterus spook* (*spo*), *shade* (*shd*), *Ecdysone receptor* (*EcR*), *ultraspiracle* (*usp*), *Ecdysone*-*induced protein 75* (*E75*), *Hormone receptor 51* (*Hr51*), *Hormone receptor* 3 (*Hr3*), *abrupt* (*ab*), and *yorkie* (*yki*) were uploaded to GenBank under accession numbers: OQ606003 (*ab*), OQ606004 (*E75*), OQ606005 (*EcR*), OQ606006 (*Hr3*), OQ606007 (*Hr51*), OQ606008 (*shd*), OQ606009 (*spo*), OQ606010 (*usp*), OQ606011 (*yki*).

### *Taiman* gene models

***Pyrrhocoris apterus taiman* gene model** was built according to [64] in Geneious Prime 2022 (Biomatters, Auckland, New Zealand). Briefly, *taiman* transcripts were identified as all other genes (see Gene identification, cloning, RNA interference) and *taiman* coding sequence was used for searching for full-length cDNA reads, generated from polyA+ mRNAs from Oxford Nanopore Technology (ONT) sequencing. ONT reads were then mapped to in-house genomic *P*. *apterus* assembly (which will be published elsewhere) and corresponding *taiman* messenger RNA and coding sequences were manually annotated in the *taiman* genomic locus. The resulting gene model, including messenger RNA and coding sequence isoforms, is deposited in GenBank under accession number OQ606002. *Taiman* ONT transcriptomic reads from brain transcriptomes (female Long Day (LD) 20°C; female LD 25°C; female LD 32°C; male LD 20°C; male LD 25°C; male LD 32°C; female Short Day (SD) 25°C; male SD 25°C) were mapped to *tai* locus using Minimap2 mapper (in Geneious Prime 2022), and reads unequivocally mapped to picked *tai* exons were manually counted and used for *tai* isoform/exon semi-quantitative expression ration calculation. Reads mapped to exons number 1, 2, and 3, respectively, were used to estimate *tai* starting exon preference. To assess *tai* alternative splicing at the 3’ end, only ONT brain reads mapped to exon 19 were collected and mapped to the exon 20 extension (representing *tai*-C, F transcripts) and exon 21 (*tai* iso-A, D, G), respectively. Reads unmapped to exon 20 extension and exon 21 were counted as representing isoforms *tai* iso-B, E, H.

***Blattella germanica taiman* gene model** was built using overlapping genomic sequences PYGN01000796.1 and JPZV02018570.1, which were assembled, and transcripts HG965205.1

HG965206.1, HG965207.1, and HG965208.1 [46] were mapped to the resulting genomic assembly. Mapping unraveled that all four transcripts encode an extra 30 amino acids at the very 5’ end.

### Larval and adult injections

#### *P*. *apterus* injections

All 1-2-day-old *P*. *apterus* adult males were injected with a Hamilton syringe (Hamilton) or 10-μl micro syringe (VWR/Avantor) with 2 μl of 3–4 μg/μl dsRNA dorsolaterally into the abdomen, at Zeitgeber Time = 3–5. Two-day-old *P*. *apterus* adult females, and one-day-old fourth- and fifth-instar larvae were briefly CO2 anesthetized, attached to the double stick tape on a tray and ventrolaterally injected into the abdomen under the stereomicroscope using a micromanipulator (Narishige, Japan) holding a borosilicate glass capillary needle. Each nymph was injected with circa 1 μl of 3–4 μg/μl dsRNA, and each adult female with 2 μl of 3–4 μg/μl dsRNA.

Injected adult males were either loaded into Locomotor Activity Monitors for measuring the free-running period (see Locomotor activity measurement) or crossed to intact females two days after injection for assessing the paternal effect on female reproduction. Females were crossed to intact adult males two days after the injection and scored for the egg laying onwards. Injected larvae and all adult bugs (except those for Locomotor activity measurement) were transferred to glass jars supplemented with linden seeds and water. Fourth-instar larvae were scored for survival and the number of ecdysis events during the rest of their development. Seven-day-old fifth-instar larvae, freshly hatched adult males, and seven-day-old adult males were collected for makisterone A measurement at Zeitgeber Time = 3–5.

#### *B*. *germanica* injections

Adult males were anesthetized by CO_2_ and injected ventrolaterally with a 10-μl Hamilton syringe (Hamilton) with 1 μl of 3–4 μg/μl dsRNA. Males were then transferred to 0.5-liter jars supplemented with bread and water *ad libitum* for four days (under 16 h light/8 h dark) before being loaded into Petri dishes for locomotor activity measurement (see below).

### Administration of hormones and allatectomy in *Pyrrhocoris apterus*

Adult males were anesthetized by CO2 and topically treated by 4 μl of 0.1 μg/μl methoprene (VUOS Pardubice, Czech Republic) dissolved in acetone. Control males were treated with acetone or were not treated at all (intact males). Ecdysteroids, ecdysone mimic, and control ethanol solutions were injected into the male bug abdomen: 2 μl of 0.45 μg/μl 20-hydroxy-ecdysone (20E, 98% purity, 20E was extracted from the plant *Rhaponticum carthamoides* and gifted to us by Dr. P. Šimek, Biology Centre CAS) in 5% ethanol; 2 μl of 0.5 μg/μl makisterone A (Abcam, Cambridge, United Kingdom); 2 μl of 0.5 ng/μl or 2 μl of 5 ng/μl of ecdysone mimic ponasterone A (Abcam.) were injected into the abdomen of male bugs by Hamilton syringe (Hamilton). For each hormone, control animals were treated by injecting the mock buffer or were not treated at all (intact males).

Allatectomies were performed as previously published [65,66]. Briefly, male individuals were anesthetized by submerging in water for 10 minutes and *corpus allatum* was removed through an incision in the neck membrane, control (= sham) animals were only cut in the neck membrane.

### Makisterone A measurements in *Pyrrhocoris apterus*

#### Derivatization

Standard solutions of makisterone A as well as the samples were derivatized [67] with 1 ml of 100 mg/ml hydroxylamine hydrochloride aqueous solution for 90 min at 70°C. Analytes were extracted by 2 x 1.5 ml of methyl tert-butyl ether. The organic phase was evaporated to dryness and reconstituted in 100 μl of 1% formic acid in methanol.

#### Quantitative analysis by ultra-high-performance liquid chromatography-triple quadrupole mass spectrometry

UHPLC was performed on Exion LC system with Kinetex 1.7 um C18 100x 2.1 mm column with 0.2 ml/min flow of mobile phase (0.1% CH3COOH in H2O / 0.1% CH3COOH in acetonitrile). LC system was connected to triple quadrupole mass spectrometer SCIEX QTRAP 6500+ system with ESI ion source. Data processing was performed in quantification Analyst Software by Sciex.

### Locomotion activity monitoring

#### P. apterus

Males were individually housed in test tubes (2.5 cm diameter, 15 cm in length) directly after the treatment (dsRNA injection; methoprene, 20-hydroxy-ecdysone, makisterone A, ecdysteroid mimic ponasterone A, acetone (control), ethanol (control) application; allatectomized males; intact males), supplemented with dry linden seeds and water *ad libitum* and placed in the Locomotor Activity Monitors (LAM 25, TriKinetics Inc., Waltham, MA) in the incubators with a constant temperature of 25°C. For 5 days, bugs were kept in LD conditions (18 h light, 6 h darkness) with the illumination of 500 lux followed by 10–15 days in constant darkness (DD). The free running period (FRP, τ) was determined for each individual as previously described [63].

#### B. germanica

Males were injected with 1 μl of *tai* fragment #1 or #2 dsRNA or control *lacZ* dsRNA and individually placed in Petri dishes partially filled with agar, providing food and water. Males in Petri dishes (44–47 dishes run in parallel) were loaded into a chamber with a controlled light regime (16 h light/8 h dark for four days, then constant dark), temperature (27°C ± 0,5°C), magnetic field corresponding to a typical geomagnetic field intensity (30 μT ± 0,6 μT), and constant sound noise. Two Infra-Red (850 nm) LED spotlights illuminated dishes from below and enabled males in Petri dishes to be recorded from above by a camera (DMK 31AU03, The Imaging Source, Charlotte, North Carolina, United States). Single frames were saved to a connected computer every 5 minutes using IC Capture software (The Imaging Source, see above). A layer mask of Petri dishes was made in GNU Image Manipulation Program (GIMP, freeware). The mask and frames were loaded and automatically analyzed in Matlab-based custom-made image analysis software (available on-line at https://is.muni.cz/www/vacha/roachlab_sw/). Briefly, every frame was scored for male body shift greater than 1 cm, and the resulting text file was analyzed by the Lomb–Scargle method in the ActogramJ plugin of ImageJ software (1.49v; NIH) with P < 0.01. Only males with PN values ≥20 and with one clear peak of locomotor activity were counted as strong rhythmicity males. Complex rhythmicity males had more than one peak of period of their free-running locomotor activity, or their free-running period changed during the measurement. Males with PN values <20 were considered as arrhythmic.

### Statistics

All statistical analyses were determined in Prism 7 (GraphPad Software, La Jolla, CA, USA). One-way ANOVA with Dunnett’s multiple comparisons post hoc test was used for all locomotor activity analyses except for the comparison of control (intact) and *Met* dsRNA, and intact vs. *E75* fr #2, where an unpaired two-tailed *t*-test was used. Makisterone A peak area of each sample is an average of three technical replicates. The peak area mean values (= biological measurements) were logarithmized with a common logarithm and the statistical difference was calculated using One-way ANOVA with Tukey’s multiple comparisons post hoc test in Prism 7.

## Supporting information

S1 Fig Makisterone A level in *P*. *apterus* males and larvae. Makisterone A peak areas corresponding to the reference makisterone A peak were determined by ultra-high-performance liquid chromatography-triple quadrupole mass spectrometry and normalized to the body mass. Statistical differences between treated groups were determined by One-way ANOVA with Tukey’s multiple comparisons post hoc test (*** p < .001). ns = statistically nonsignificant (p > .05). Adult males (n = 4), fifth-instar larvae (n = 3). Y-axis is on a logarithmic scale.
(EPS)

S1 Table FRP values of *P*. *apterus* males with strong rhythmicity in Fig 2. (XLSX)

S2 Table FRP values of *P*. *apterus* males with strong rhythmicity in Fig 4. (XLSX)

S3 Table FRP values of *B*. *germanica* males with strong rhythmicity in Fig 7. (XLSX)

S4 Table Fig 2 statistics. (XLSX)

S5 Table Fig 4 statistics. (XLSX)

S6 Table Fig 7 statistics. (XLSX)
